# Stage-specific *Plasmodium falciparum* immune responses in afebrile adults and children living in the Greater Accra Region of Ghana

**DOI:** 10.1186/s12936-020-3146-7

**Published:** 2020-02-10

**Authors:** Festus K. Acquah, Aminata C. Lo, Kwadwo Akyea-Mensah, Hamza B. Abagna, Babacar Faye, Michael Theisen, Ben A. Gyan, Linda E. Amoah

**Affiliations:** 1 Immunology Department, Noguchi Memorial Institute for Medical Research (NMIMR), University of Ghana, Accra, Ghana; 2 West African Centre for Cell Biology of Infectious Pathogens (WACCBIP), University of Ghana, Accra, Ghana; 3 Present Address: Parasitology Department, University Cheikh Anta Diop, Dakar, Senegal; 4 Parasitology Department, University Cheikh Anta Diop, Dakar, Senegal; 5 Department for Congenital Disorders, Statens Serum Institut, Copenhagen, Denmark; 6 Centre for Medical Parasitology at Department of International Health, Immunology and Microbiology, University of Copenhagen, Copenhagen, Denmark

**Keywords:** Transmission, Gametocyte, Afebrile, Antibody

## Abstract

**Background:**

Asymptomatic carriage of *Plasmodium falciparum* is widespread in adults and children living in malaria-endemic countries. This study identified the prevalence of malaria parasites and the corresponding levels of naturally acquired anti-parasite antibody levels in afebrile adults living in two communities in the Greater Accra Region of Ghana.

**Methods:**

Two cross-sectional studies conducted in January and February 2016 and repeated in July and August 2016 recruited subjects aged between 6 and 75 years from high parasite prevalence (Obom) and low parasite prevalence (Asutsuare) communities. Whole blood (5 ml) was collected from each volunteer, plasma was aliquoted and frozen until needed. An aliquot (10 µl) of the blood was used to prepare thick and thin blood smears, 100 µl was preserved in Trizol and the rest was separated into plasma and blood cells and each stored at − 20 °C until needed. Anti-MSP3 and Pfs230 antibody levels were measured using ELISA.

**Results:**

Asexual parasite and gametocyte prevalence were higher in Obom than Asutsuare. Antibody (IgG, IgG1, IgG3, IgM) responses against the asexual parasite antigen MSP3 and gametocyte antigen Pfs230 were higher in Obom during the course of the study except for IgM responses against Pfs230, which was higher in Asutsuare than in Obom during the rainy season. Antibody responses in Asutsuare were more significantly associated with age than the responses measured in Obom.

**Conclusion:**

The pattern of antibody responses measured in people living in the high and low malaria transmission setting was similar. All antibody responses measured against the asexual antigen MSP3 increased, however, IgG and IgG1 responses against gametocyte antigen Pfs230 decreased in moving from the dry to the peak season in both sites. Whilst asexual and gametocyte prevalence was similar between the seasons in the low transmission setting, in the high transmission setting asexual parasite prevalence increased but gametocyte prevalence decreased in the rainy season relative to the dry season.

## Background

Asymptomatic carriage of malaria parasites has been associated with the development of immunity to malaria mainly due to continuous exposure of the host’s immune system to the *Plasmodium* parasites [1]. Low parasite density in low transmission settings as well as frequent exposure to similar parasite isolates in high transmission settings have been suggested to enhance the establishment of asymptomatic infections [2]. The level of exposure and rate at which antibodies against both the asexual and sexual stage (gametocytes) parasites are acquired and boosted may be different for people living in different malaria transmission settings [1].

The propagation of *Plasmodium* parasites within the human erythrocyte, which is critical for the survival of the parasite is initiated by the merozoite. The merozoite is one of several daughter cells released from a mature schizont, which proceed to invade a new host erythrocyte and continue the asexual erythrocytic cycle of the parasite [3]. Merozoites are not contained within erythrocytes and as such their surface antigens are exposed directly to the host’s immune system. A number of antigens expressed on the surface of the *Plasmodium falciparum* merozoite, including the merozoite surface protein 1 (MSP1) and 3 (MSP3) have been validated as malaria vaccine candidates [4, 5] due to their ability to induce protective antibodies against malaria. Antibodies specific to MSP3 exert anti-parasite effects, either through inhibition of the merozoite invasion in erythrocytes or in cooperation with mononuclear cells through antibody-dependent cellular inhibition and opsonic phagocytosis [6, 7]. During the erythrocytic cycle, some of the asexual parasites develop into sexual forms: gametocytes. Antigens, including Pfs230 and Pfs48/45, which are expressed during gametocytogenesis have been found to be immunogenic [8, 9]. Pfs230 is a gamete surface antigen [10–12] and marked as a transmission blocking vaccine candidate [13]. Antibodies against Pfs230 have been detected in populations naturally exposed to malaria parasites [14, 15]. Such antibodies together with specific antibodies generated in small rodents have been shown to inhibit parasite development in the standard membrane-feeding assay (SMFA) considered the ‘gold standard’ assay for functional transmission-blocking antibodies [16–18]. These antibodies, however, have been suggested to be very short lived, peaking during the transmission season [19] and are more prevalent in children than in adults [15].

Immunoglobulin G (IgG) antibodies have been shown to be a very important component of humoral immunity in the fight against *Plasmodium* infections as they have associated with protections against infection [20–22] and transmission-reducing immunity [23, 24]. Cytophilic antibodies (IgG1 and IgG3) have been shown to be particularly important in anti-malarial immunity and associated with protection from the disease [25–29]. Monitoring antibody responses in asymptomatic individuals is thus a valuable tool for monitoring the acquisition of anti-disease immunity as well as the frequency and magnitude of parasite infection [1]. A few earlier studies have characterized natural antibody responses to both asexual and sexual stage antigens, however these studies have only looked into immune responses in asymptomatic children below the age of 12 years [15] or in a symptomatic population [30]. Other studies on afebrile individuals have characterized antibody responses against sporozoite [31], asexual [32, 33] or only sexual stage [34] antibody responses amongst a cohort of Ghanaians. This current study goes further to compare the characteristics of naturally acquired immune responses to asexual parasite antigen MSP3 and sexual stage antigen Pfs230 in both afebrile adults and children living in two communities with different malaria parasite prevalence and transmission intensities.

## Methods

### Ethics statement

The Institutional Review Board (IRB) of Noguchi Memorial Institute for Medical Research granted ethical approval for the study (Study number 089/14-15). Written informed consent was obtained individually from all participants before they were enrolled into the study.

### Study site

**Fig. 1:**
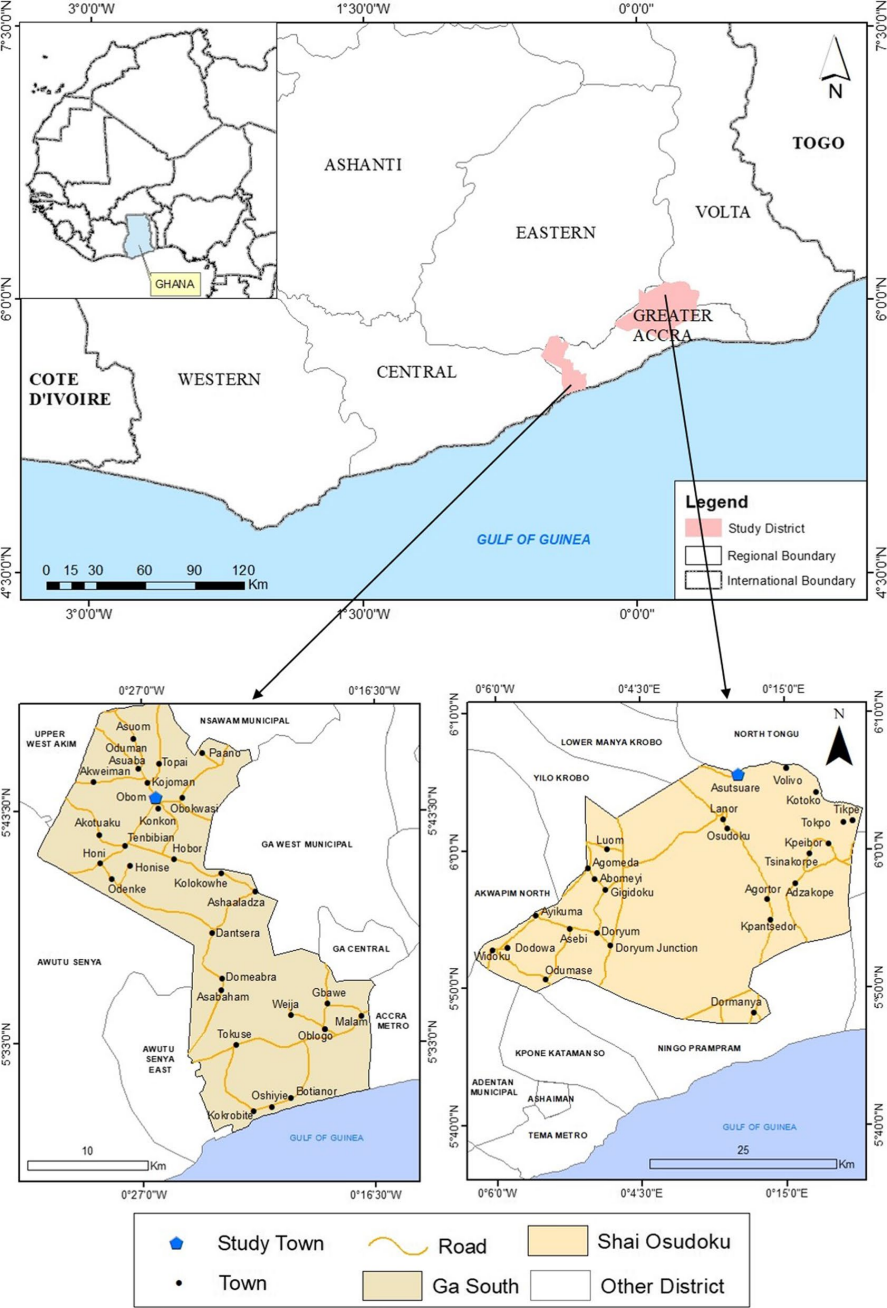
A map of Ghana projecting study sites located within the Greater Accra Region. The map was created using shapefiles from the Survey Department of the Ghana Statistical Services and ArcMap GIS v10.5 (no administrative permissions were needed to access the shapefiles). Courtesy Mr Richard Adade, GIS and Remote Sensing Unit, Department of Fisheries and Aquatic Sciences, Centre for Coastal Management, University of Cape Coast, Cape Coast, Ghana

This study was carried out in Obom and Asutsuare (Fig. 1), both in the Greater Accra Region of Ghana [35]. Obom is a semi-rural community in the Ga South Municipality. Malaria transmission in Obom is perennial, although it has a peak transmission period from May to September. According to ongoing mapping studies in the community, malaria parasite prevalence in Obom was estimated at 41% during the peak transmission period in 2014 [15]. Asutsuare is a semi-rural community located in the Shai-Osudoku district of Dangme West Municipality. Malaria transmission is low but perennial, and peaks slightly during and immediately after the major rainy season (April to July) and is lowest during the dry season [36].

### Sample collection

Volunteers aged between 6 and 75 years old and without any symptom of malaria were recruited into a cross-sectional study in February 2016 (dry season), which included 264 volunteers from Obom and 230 volunteers from Asutsuare. In July 2016 (rainy season) a second set of volunteers was recruited, 192 from Obom and 174 from Asutsuare, which included 120 volunteers from the off-peak season sample collection for both sites. Venous blood (5 ml) was collected from each volunteer into acid dextrose vacutainer tubes on the day of recruitment. An aliquot (10 µl) of the blood was used to prepare thick and thin blood smears, 100 µl was preserved in Trizol and the rest was separated into plasma and blood cells via centrifugation and independently stored at − 20 °C until used.

### Parasite detection

The thin and thick blood smears were processed and used to identify and quantify the presence of *P. falciparum* parasites [37]. Briefly, thin smears were air dried, fixed in 100% methanol and then stained for 15 min in a 10% Giemsa stain solution. Thick smears were air dried and stained in 10% Giemsa for 15 min. Both set of slides were subsequently observed using 100× oil immersion microscope. Parasite density was estimated based on the total number of parasites counted per 200 white blood cells (WBCs). Two independent microscopists read the stained slides.

### Gametocyte detection

RNA was isolated from the Trizol-preserved whole blood using the Quick RNA miniprep kit (Zymo Research, USA) according to manufacturer’s instructions and subsequently converted into cDNA using the Protoscript II first strand cDNA synthesis kit (NEB, UK) as previously described [38]. Genomic DNA contamination of each extracted RNA was assessed as previously described [39]. A semi-quantitative Pfs25 mRNA RT-PCR method similar to that described by Ayanful-Torgby et al. [39, 40] was used to determine gametocyte prevalence. Briefly, 300 nM of Pfs25 F and R primers were added to 2 ul of cDNA (1:10) and fast SYBR^®^ Green 2X master mix RT-PCR kit (Applied Biosystems). The reactions were run in triplicate on a QuantStudio 3™ Real-Time PCR System (Thermo Scientific, USA). The primer validation and all positive and negative controls used in this experiment have been previously described [39, 40].

### Enzyme-linked immunosorbent assay (ELISA)

Antibody responses including IgG, IgM, IgG1, and IgG3 against recombinant *P. falciparum* sexual stage and asexual stage antigens were quantified using an indirect ELISA protocol [41]. The antigens used in this study include Pfs230 [14] and MSP3 [30] produced in *Lactococcus lactis*. Briefly Pfs230 antigen was diluted to 1 µg/ml in carbonate buffer [14, 15] and MSP3 diluted to 1 µg/ml in phosphate-buffered saline (1X PBS, pH7.2) [15, 30] and 100 µl/well of the diluted antigen was used to coat the wells of Maxisorp NUNC plates (Nunc Maxisorp, UK) overnight at 4 °C. The plates were subsequently washed with wash buffer (PBST; 1X PBS supplemented with 0.05% Tween 20 at pH7.2), blocked with 3% non-fat skimmed milk (Marvel, UK) in1X PBS and incubated at room temperature (RT) for 1 h. The plates were then incubated with 100 µl/well of plasma diluted to 1:200 for IgG and IgM and 1:100 for IgG1 and IgG3 in 1% of non-fat skimmed milk in 1X PBS. Two pools of sera, one previously determined to have high concentrations of antibodies against MSP3 and the other against Pfs48/45, were used separately as a standard calibrator. The standards were used at a starting dilution of 1:400 for IgG and IgM and 1:100 for the cytophilic sub-classes IgG1 and IgG3 and serially diluted two-fold for an additional seven concentrations. Plasma samples were incubated for 1 h at RT for IgG and IgM and 37 °C for IgG1 and IgG3. Each plate was washed three times with wash buffer after every incubation step. The plates were subsequently incubated with 100 µl/well of goat anti-human IgG or IgM at a 1:3000 dilution or 100 µl/well of goat anti-human IgG1 or IgG3 at a dilution of 1:1000 for 1 h at RT, followed by a final wash step. The plates were then incubated with 50 µl/well of TMB plus2 for 15 min. Colour development was stopped by the addition of 50 µl/well of 0.2 M H_2_SO_4_ and optical densities (OD) read at 450 nm using a 96-well ELISA plate reader (Biotek, VT, USA).

### Statistical and data analysis

The cut-off for positivity for gametocyte presence by RT-PCR as determined by the no template control was ‘undetermined’. Any sample that yielded a CT value other than ‘undetermined’ was scored as positive.

For each measured antibody, OD data were normalized against the standard calibrator. OD data were converted into concentration in arbitrary units using the 4-parameter logistic curve-fitting program, ADAMSEL (version b040; Ed Remarque™). Log10-transformed OD data from naïve malaria volunteers from the two seasons were used to define a common cut-off from which seroprevalence was calculated as the population of sample with Log10-transformed ODs higher than the common cut-off. Analysis of data and graphics was performed using Kruskal–Wallis test, Spearman correlation and other statistical tests (Graph Pad Prism version 7). Statistical significance was set as P ≤ 0.05, unless otherwise stated.

## Results

### Study participants

The study recruited 230 and 174 volunteers from Asutsuare during the dry (January–February) and rainy (July–August) seasons, respectively, and 264 and 192 volunteers from Obom during the dry (January–February) and rainy (July–August) seasons, respectively. Volunteers aged 10 years and below were the least represented in both sites at both sampling time points, and participants aged above 15 years were the most represented in Asutsuare at both sampling time points (Table 1). Age data were not captured for some of the samples, making the sum of samples analysed in the three cohorts (age-stratified data) less than the total number of samples recruited. Gametocyte prevalence by microscopy was very low in both communities, with the prevalence ranging between 0.4 and 0.6%. Asexual stage parasite prevalence and density was much higher in Obom than in Asutsuare during both the dry and rainy seasons (Table 1). Whilst there was an increase in the prevalence of asymptomatic parasite carriers from the dry to the rainy season in Obom, asymptomatic parasite prevalence in Asutsuare remained relatively the same at the two time points (Table 1).

**Table 1:** Demographic characteristics and parasitological indices of study participants

	Obom		Asutsuare	
	Dry (264)	Rainy (192)	Dry (230)	Rainy (174)
Age/years				
≤ 10 years (N(%))	36 (13.6)	35 (18.2)	22 (9.6)	18 (7.8)
11–15 years (N(%))	117 (44.3)	78 (40.6)	58 (25.2)	42 (24.1)
≥ 16 years (N(%))	101 (38.3)	73 (38.0)	141 (61.3)	113 (64.9)
Asexual carriers	32 (12.1)	62 (32.3)	8 (3.5)	7 (4.0)
PD (p/µl)				
Min–Max	16–5080	16–4219	40–280	16–1553
Median (IQR)	120 (80–350)	112 (48–528)	40 (40–170)	400 (32–955)
Gametocyte carriers	47 (17.8)	30 (15.6)	3 (1.3)	3 (1.7)

*N* number in counts, *p/µl* parasites per µl of blood, *Min* minimum, *Max* maximum, *IQR* interquartile range, *PD* parasite density

The sites are listed with the number of study participants in brackets. The values in the table represent frequency as counts (N) and % in brackets of the total population at each site during each time point. Study participants were stratified into three age groups, ≤ 10 years, 11–15 years and ≥ 16 years. *N* number of people. Asexual parasite prevalence was detected by light microscopy and gametocyte prevalence by Pfs25 mRNA RT-PCR

### Asexual antibody responses

**Fig. 2:**
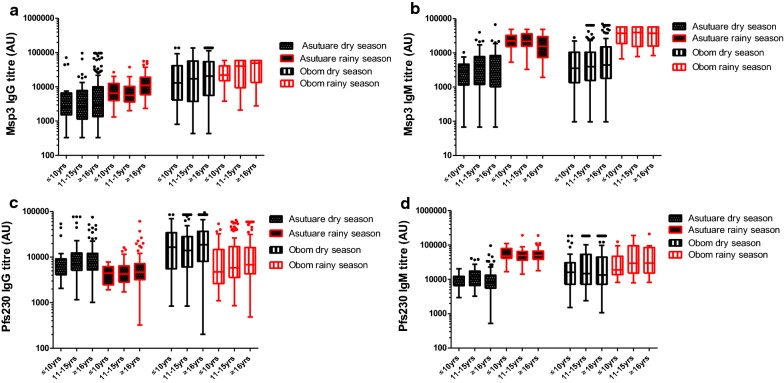
Age-stratified IgG and IgM responses. Antibody responses: IgG responses against MSP3 (**a**) and Pfs230 (**c**) and IgM responses against MSP3 (**b**) and Pfs230 (**d**) in the study participants were stratified into children 10 years old and below (≤ 10 years), children between 11 and 15 years (11–15 years) and adults 16 years and above (≥ 16 years). Measurements were made in both the dry and rainy season from Obom and Asutsuare. Antibody concentrations are presented in arbitrary units (AU) on a Tukey box-and-whiskers plot

**Fig. 3:**
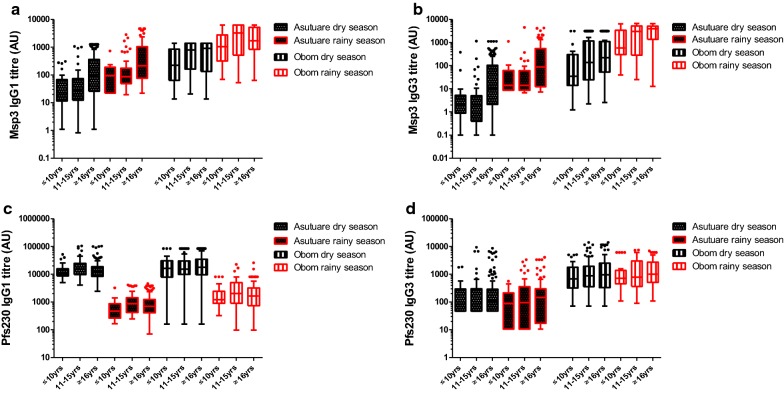
Age-stratified IgG1 and IgG3 responses. IgG1 responses against MSP3 (**a**) and Pfs230 (**c**) and IgG3 responses against MSP3 (**b**) and Pfs230 (**d**) in the study participants were stratified into children 10 years old and below (≤ 10 years), children between 11 and 15 years (11–15 years) and adults 16 years and above (≥ 16 years). Measurements were made in both the dry and rainy season from Obom and Asutsuare. Antibody concentrations are presented in arbitrary units (AU) on a Tukey box-and-whiskers plot

At the community level, total IgG for naturally induced antibodies against MSP3 increased significantly in the rainy season compared to the dry season for both sites (Fig. 2a), with the increase in Asutsuare being greater than in Obom (Kruskal–Wallis test, p < 0.001 in Asutsuare and p < 0.01 in Obom). Total IgG levels in Obom were significantly higher than in Asutsuare at all time points (Kruskal–Wallis test, p < 0.05, Additional file 1: Table S1). A similar trend was observed for naturally induced IgM, IgG1 and IgG3 antibodies against MSP3 (Figs. 2b and 3a, b), where all responses measured in Obom were significantly higher than those recorded in Asutsuare (Additional file 1) and the rainy season having higher levels compared to the dry season (Figs. 2a, b and 3a, b).

During the dry season, age was found to correlate with IgG1 as well as IgG3 responses against MSP3 in both Obom and Asutsuare. In Obom, the correlation was significant but weak; Spearman r = 0.1514, p = 0.0228 (IgG1) and Spearman r = 0.2633, p = 0.0001 (IgG3), whilst in Asutsuare, the correlations although higher than Obom were still weak; Spearman r = 0.3341 (IgG1), Spearman r = 0.4057 (IgG3), p < 0.0001 for both) (Additional file 1).

Significant differences in IgG1 as well as IgG3 responses against MSP3 amongst the three age groups at each site were noted (Fig. 3a, b). IgG1 responses against MSP3 in Obom were significantly lower (Dunn’s Multiple Comparison Test, p < 0.05) in young children (≤ 10 years) than in the older children (11–15 years). The measured IgG3 responses against MSP3 in the young children (≤ 10 years) were significantly lower (Dunn’s Multiple Comparison Test, p < 0.01) than responses measured in the adults (≥ 16 years) (Fig. 3b).

In Asutsuare, IgG1 responses against MSP3 (Fig. 3a) were significantly higher in the adults (≥ 16 years) compared to both young children (≤ 10 years) and the older children (11–15 years) (Dunn’s Multiple Comparison Test, p < 0.01 and 0.001, respectively). A similar observation was made for anti-MSP3 IgG3 responses where the adults had significantly higher responses than both young children and older children (Dunn’s Multiple Comparison Test, p < 0.05 and 0.001, respectively).

During the rainy season, total IgG (Spearman r = 0.2027, p = 0.0207) (Fig. 2a) and IgG3 (Spearman r = 0.2449, p = 0.0059) against MSP3 in volunteers from Obom showed a positive but weak correlation with age. In Asutsuare, total IgG (Spearman r = 0.3672, p < 0.0001), IgG1 (Spearman r = 0.3962, p < 0.0001) and IgG3 (Spearman r = 0.4485, p < 0.0001) responses against MSP3 (Fig. 3a, b) significantly correlated weakly but positively with age. Anti-MSP3 IgM antibodies correlated inversely with age in Asutsuare (Spearman r = − 0.1788, p < 0.05) but not in Obom (Fig. 2b).

Anti-IgG3 responses measured in Obom against MSP3 remained significantly lower in the young children (≤ 10 years) compared to the adults (≥ 16 years) (Dunn’s Multiple Comparison Test, p < 0.05), however, IgG, IgG3 and IgM levels across the three age groups were similar during the rainy season (Figs. 2a, b and 3a, b). In Asutsuare, anti-IgG1 and IgG3 responses measured against MSP3 was significantly higher in adults than those measured in the young and older children (Dunn’s Multiple Comparison Test, p < 0.05 (young children) and p < 0.001 (older children) for both). Total IgG responses were significantly higher in adults (Dunn’s Multiple Comparison Test, p < 0.001) than in older children, whilst no differences in IgM responses were measured between the three groups (Dunn’s Multiple Comparison Test, p > 0.05 for all combinations).

Contribution of the measured cytophilic antibodies to variations in measured total IgG was assessed by fitting a linear regression model using IgG1 and IgG3 as explanatory variables for total IgG concentrations against MSP3. The results showed that in the dry season, the independent variables (anti-IgG1 and IgG3) could not explain the variations observed in total IgG responses in Obom and could only account for 3.3% of the variations in total IgG observed in Asutsuare (Table 2). In the rainy season however, about 70% (for Asutsuare) and 71% (for Obom) of the variations in total anti-MSP3 IgG could be explained by variations in the measured IgG1 and IgG3 (R^2^ = 0.699, p < 0.001 and R^2^ = 0.709, p < 0.001 for Asutsuare and Obom, respectively). All variance inflation factors (VIFs) for all the analyses were less than 1.8 (Additional file 2).

**Table 2:** Multivariate linear regression relating total IgG with IgG1 and IgG3

Independent variables	IgG1 and IgG3	
	R^2^	P
MSP3		
Asutsuare (dry)	0.033	0.018
Asutsuare (rainy)	0.699	< 0.001
Obom (dry)	− 0.006	0.426
Obom (rainy)	0.709	< 0.001
Pfs230		
Asutsuary (dry)	0.455	< 0.001
Asutsuary (rainy)	0.111	< 0.001
Obom (dry)	0.313	< 0.001
Obom (rainy)	0.003	0.301

*R*^*2*^ coefficient of determination; *P* p value

### Sexual stage (Pfs230) antibody responses

Within the community, all antibody responses measured against Pfs230 in Obom were significantly (Kruskal–Wallis test, p < 0.001) higher than that measured in Asutsuare. Moving from the dry to the rainy seasons, naturally induced IgG responses against Pfs230 decreased significantly (Kruskal–Wallis test p < 0.001) (Fig. 2c), whilst anti-IgM increased significantly (Kruskal–Wallis test, p < 0.001) in both sites. Anti-IgM levels in Obom were significantly (Kruskal–Wallis test, p < 0.001) (Fig. 2d) higher than those measured in Asutsuare in the dry season but in the rainy season, IgM responses measured in Obom were significantly (Kruskal–Wallis test, p < 0.001) lower than those measured in Asutsuare (Fig. 2d). Anti-IgG1 responses measured in both sites were similar to that of total IgG, with a decrease observed in moving from the dry to the rainy season. Anti-IgG1 levels measured in Obom in the rainy season were significantly (Kruskal–Wallis test, p < 0.001) higher than measured in Asutsuare in the same season but anti-IgG1 responses measured in the dry season were similar at both sites (Fig. 3c). Anti-IgG3 responses against Pfs230 were similar between seasons in both sites (Fig. 3d).

During the dry season, age was not found to correlate with any of the Pfs230 antibody responses measured in Obom, however a significant (Spearman r = − 0.020, p = 0.0029) very weak negative correlation was observed between age and IgM responses in Asutsuare.

During the rainy season, age positively (Spearman r = 0.1718, p = 0.0172) correlated with anti-Pfs230 total IgG in volunteers from Obom, whilst age correlated significantly (Spearman r = 0.2582, p = 0.0007) with IgG3 responses in Asutsuare. Anti-IgG1 responses against Pfs230 from both sites correlated negatively with age but the correlation was not significant (Spearman r = − 0.169, p = 0.8233 for Obom and Spearman r = − 0.0571, p = 0.5011 for Asutsuare).

The weak correlation of antibody responses with age was reinforced when no significant differences were observed between all the various antibody responses measured against Pfs230 in the young children, the older children and the adults from both Obom and Asutsuare during both the dry and rainy season (Figs. 2c, d and 3c, d). Similar to the asexual stage antibodies against MSP3, variations in measured anti-IgG1 and IgG3 against Pfs230 could not account for the variations in IgG measure in Obom but could account for 11% of the IgG variations measured in Asutsuare (Table 2). However, during the dry season, the R^2^ increased to 0.455 (p < 0.001 for Asutsuare) and 0.313 (p < 0.001 for Obom) (Table 2). All VIFs were approximately 1.

The parasite density of samples collected from Obom during both the dry and rainy season did not correlate with any of the measured antibody (asexual stage or the gametocyte) responses. There were too few samples with microscopic densities of parasites in Asutsuare to do any statistical analysis (Additional file 3).

## Discussion

Asymptomatic carriage of *P. falciparum* parasites exposes the host to both asexual disease-causing parasites as well as the sexual transmissible forms of the parasite. This primes as well as boosts the host’s immune system to produce antibodies against both asexual and sexual (gametocyte) forms of the parasite. Inhabitants of malaria-endemic countries, especially children, are highly at risk of being infected with the malaria parasite and have previously been the focus of earlier studies to understand and identify differences in asymptomatic parasite carriage in high and low malaria transmission communities in Ghana [15, 40]. In this study, afebrile adults and children were recruited from communities of varying malaria transmission intensities and parasite prevalence to enable the comparison of naturally acquired immune responses against asexual and sexual stage antigens in both the dry and subsequent rainy season.

As anticipated, the prevalence of asymptomatic carriers was significantly higher in Obom, the high parasite prevalence setting, than in Asutsuare where *P. falciparum* parasite prevalence has been reported to be very low for over a decade [34, 36]. No significant difference was observed in asexual parasite carriage between the dry and rainy season in Asutsuare, mainly because malaria is low and perennial [36] and supports an earlier report on asexual parasite prevalence in another low malaria parasite intensity setting of Ghana [40]. However, there was an almost two-fold increase in microscopic levels of asexual parasite carriage in moving from the dry to the rainy season in Obom, where malaria is high and seasonal. Gametocyte carriage in Obom significantly reduced in moving from the dry to the rainy season. A similar finding of reduced gametocyte carriage in the peak relative to the off-peak season has been reported in young children from Obom [40]. The absence of variation in gametocyte carriage across the dry and rainy season in Asutsuare is supportive of the very low year-round malaria transmission recorded in Asutsuare. The low gametocyte prevalence identified in the participants from Asutsuare supports a recent report that identified low gametocyte prevalence amongst children and adults, including pregnant women in Asutsuare [34].

A significant increase in the community-wide levels of anti-MSP3 IgM was anticipated and confirmed in moving from the dry to the rainy season in Obom, where there was a subsequent increase in asexual parasite prevalence. Interestingly, a similar significant increase in anti-MSP3 IgM was observed in Asutsuare, although asymptomatic carriage of microscopic densities of *P. falciparum* parasites remained the same in moving from the dry to rainy season. One possible explanation for the increase in IgM in rainy season could be an increase in the prevalence of sub-microscopic density infections in the rainy season that was not captured by microscopy but has been reported using more sensitive tools [42]. In Asutsuare, anti-MSP3 IgG did not correlate with age, however the levels in adults during the rainy season were higher than in the children. It is not clear why no age correlation was observed, however, IgG antibody levels have not always been found to correlate with age [43].

Cytophilic (IgG1 and IgG3) responses against MSP3 in Asutsuare were similar in both the dry and rainy season, where the adults had significantly higher levels than both groups of children. This result suggests that more frequent exposure is likely to be required to mature cytophilic antibody responses [7], buttressing the results observed in Obom, where the cytophilic IgG responses were lower in the young children compared with the older children and adults. Multivariate linear regression of the total IgG concentration using the IgG1 and IgG3 concentrations revealed that in the rainy season, IgG1 and IgG3 concentrations accounted for most of the measured total IgG. The differences observed in the rainy season relative to the dry season could be due to the increase in the prevalence of asexual parasites during the rainy season relative to the dry season since IgG1 and IgG3 are known to be potent activators of complement and phagocytic cells [44].

The reduction in the levels of Pfs230 IgG in moving from the dry to the rainy season observed in both Obom and Asutsuare could be due to a reduction in the number of gametocyte carrier, especially in Obom were fewer participants with active gametocyte infections were identified in the rainy relative to the dry season. The higher levels of Pfs230 IgM observed in Asutsuare relative to Obom in the rainy season could be due to very recent gametocyte infections in some participants in Asutsuare as antibody responses to gametocyte antigens have been suggested to develop rapidly after exposure [45]. Similarly, the high IgM levels in the rainy season could have helped with antibody clearance of the mature gametocytes from circulation, thereby causing a reduction in the detected levels gametocyte in the rainy season.

The decrease in anti-Pfs230 IgG and IgG1 with increased exposure was not unexpected as antibody responses to gametocyte antigens have been suggested to be influenced more by recent exposure compared with cumulative exposure [17]. Although IgG1 antibodies are known to have a longer half-life than IgG3 [46], the relatively unchanged levels of IgG3 but significantly decreased levels of IgG1 suggests that IgG1 might be the preferred IgG subclass required to clear mature gametocytes from circulation although further studies must be done to ascertain this.

## Conclusion

The pattern of antibody responses measured in people living in the high and low malaria transmission setting was similar. All antibody responses measured against the asexual antigen, MSP3 increased, however, IgG and IgG1 responses against gametocyte antigen Pfs230 decreased in moving from the dry to the peak season in both sites likely due to requirement of IgG1 to clear gametocytes from circulation. Whilst asexual and gametocyte prevalence was similar between the seasons in the low transmission setting, in the high transmission setting asexual parasite prevalence increased but gametocyte prevalence decreased in the rainy season relative to the dry season.

## **Supplementary information**

**Additional file 1.** Statistical analysis of antibody responses obtained at each site during both the dry and rainy season.

**Additional file 2.** Regression analysis of antibody responses obtained at each site during both the dry and rainy season.

**Additional file 3.** Correlation analysis of antibody responses obtained at Obom with age and parasite density.

## Data Availability

All data generated or analysed during this study are included in this published article [and its Additional files]

## Abbreviations

MSP3: Merozoite surface protein 3
Pfs230: Gametocyte antigen
IgG: Immunoglobulin G
IgM: Immunoglobulin M
IgG1: Immunoglobulin G subclass 1
IgG3: Immunoglobulin G subclass 3
