# Supplementary material for: Stage-specific Plasmodium falciparum immune responses in afebrile adults and children living in the Greater Accra Region of Ghana
Source: Malar J. 2020 Feb 10;19:64. doi: 10.1186/s12936-020-3146-7 (PMC7011432; doi:10.1186/s12936-020-3146-7)
Supplement: Supplementary file 1 — Additional file 1. Statistical analysis of antibody responses obtained at each site during both the dry and rainy season. [file 12936_2020_3146_MOESM1_ESM.docx]

|  | Obom Dry (AU) | Asutsuare Dry (AU) | p value | Obom Rainy (AU) | Asutsuare Rainy (AU) | p value |
| --- | --- | --- | --- | --- | --- | --- |
| MSP3 |  |  |  |  |  |  |
| IgG | 17755 (498 - 50919) | 2990 (1325 - 8000) | 0.001 | 37587 (11769 - 58760) | 8720 (4754 - 15093) | 0.001 |
| IgG1 | 617.9 (129.1 - 1369) | 50.69 (17.33 - 202.7) | 0.001 | 1944 (494.5 - 5287) | 120.9 (67.76 - 676.5) | 0.001 |
| IgG3 | 146.5 (23.66 - 801.3) | 3.94 (1.375 - 64.29) | 0.001 | 2784 (408 - 5020) | 36.87 (8.737 - 280.9) | 0.001 |
| IgM | 3981 (1564 - 10696) | 2780 (1071 - 7456) | 0.01 | 37755 (15961 - 57014) | 18790 (8949 - 31445) | 0.001 |
| Pfs230 |  |  |  |  |  |  |
| IgG | 15495 (9303 - 32376) | 7680 (5049 - 11683) | 0.001 | 5857 (3661 - 15981) | 4437 (3028 - 6757) | 0.001 |
| IgG1 | 15495 (9303 - 30864) | 12579 (8659 - 19335) | ns | 1663 (787.1 - 4199) | 714.4 (404.4 - 1309) | 0.01 |
| IgG3 | 844.4 (326.7 - 2056) | 78.75 (47.1 - 286.8) | 0.001 | 822.5 (467.3 - 2646) | 127.2 (11.9 - 298.5) | 0.001 |
| IgM | 14559 (7186 - 40555) | 9302 (5718 - 13437) | 0.001 | 25672 (14352 - 86262) | 49315 (37469 - 66066) | 0.001 |

Table S1. Statistical analysis between sites at each season

AU, arbitrary units. Values represent the median (interquartile range). The p value’s were obtained using a Dunn’s multiple comparison test between like immunoglobulin measurements obtained at both sites during the peak and off peak season.
