# Supplementary material for: Stage-specific Plasmodium falciparum immune responses in afebrile adults and children living in the Greater Accra Region of Ghana
Source: Malar J. 2020 Feb 10;19:64. doi: 10.1186/s12936-020-3146-7 (PMC7011432; doi:10.1186/s12936-020-3146-7)
Supplement: Supplementary file 2 — Additional file 2. Regression analysis of antibody responses obtained at each site during both the dry and rainy season. [file 12936_2020_3146_MOESM2_ESM.docx]

ASUTUARE V2 (DRY SEASON) – MSP3

| **Model Summary** | | | | |
| --- | --- | --- | --- | --- |
| Model | R | R Square | Adjusted R Square | Std. Error of the Estimate |
| 1 | .209^a^ | .044 | .033 | 20157.200 |

| a. Predictors: (Constant), ASv2IgG3m, ASv2IgG1m |
| --- |

| **ANOVA^a^** | | | | | | |
| --- | --- | --- | --- | --- | --- | --- |
| Model | | Sum of Squares | df | Mean Square | F | Sig. |
| 1 | Regression | 3337325330.115 | 2 | 1668662665.057 | 4.107 | .018^b^ |
|  | Residual | 72729978806.962 | 179 | 406312730.765 |  |  |
|  | Total | 76067304137.077 | 181 |  |  |  |

| a. Dependent Variable: ASv2IgGm |
| --- |
| b. Predictors: (Constant), ASv2IgG3m, ASv2IgG1m |

| **Coefficients^a^** | | | | | | | |
| --- | --- | --- | --- | --- | --- | --- | --- |
| Model | | Unstandardized Coefficients | | Standardized Coefficients | t | Sig. | Collinearity Statistics |
|  |  | B | Std. Error | Beta |  |  | Tolerance |
| 1 | (Constant) | 8499.585 | 1757.602 |  | 4.836 | .000 |  |
|  | ASv2IgG1m | 15.164 | 5.635 | .254 | 2.691 | .008 | .601 |
|  | ASv2IgG3m | -8.617 | 9.198 | -.088 | -.937 | .350 | .601 |

| **Coefficients^a^** | | |
| --- | --- | --- |
| Model | | Collinearity Statistics |
|  |  | VIF |
| 1 | (Constant) |  |
|  | ASv2IgG1m | 1.665 |
|  | ASv2IgG3m | 1.665 |

| a. Dependent Variable: ASv2IgGm |
| --- |

ASUTUARE V3 (RAINY SEASON) – MSP3

| **Model Summary** | | | | |
| --- | --- | --- | --- | --- |
| Model | R | R Square | Adjusted R Square | Std. Error of the Estimate |
| 1 | .839^a^ | .703 | .699 | 6022.151 |

| a. Predictors: (Constant), ASv3IgG3m, ASv3IgG1m |
| --- |

| **ANOVA^a^** | | | | | | |
| --- | --- | --- | --- | --- | --- | --- |
| Model | | Sum of Squares | df | Mean Square | F | Sig. |
| 1 | Regression | 12125431283.093 | 2 | 6062715641.546 | 167.172 | .000^b^ |
|  | Residual | 5113549054.734 | 141 | 36266305.353 |  |  |
|  | Total | 17238980337.826 | 143 |  |  |  |

| a. Dependent Variable: ASv3IgGm |
| --- |
| b. Predictors: (Constant), ASv3IgG3m, ASv3IgG1m |

| **Coefficients^a^** | | | | | | | |
| --- | --- | --- | --- | --- | --- | --- | --- |
| Model | | Unstandardized Coefficients | | Standardized Coefficients | t | Sig. | Collinearity Statistics |
|  |  | B | Std. Error | Beta |  |  | Tolerance |
| 1 | (Constant) | 6954.310 | 583.940 |  | 11.909 | .000 |  |
|  | ASv3IgG1m | 5.884 | .623 | .542 | 9.440 | .000 | .637 |
|  | ASv3IgG3m | 5.661 | .831 | .392 | 6.816 | .000 | .637 |

| **Coefficients^a^** | | |
| --- | --- | --- |
| Model | | Collinearity Statistics |
|  |  | VIF |
| 1 | (Constant) |  |
|  | ASv3IgG1m | 1.569 |
|  | ASv3IgG3m | 1.569 |

| a. Dependent Variable: ASv3IgGm |
| --- |

ASUTUARE V2 (DRY SEASON) – PFS230

| **Model Summary** | | | | |
| --- | --- | --- | --- | --- |
| Model | R | R Square | Adjusted R Square | Std. Error of the Estimate |
| 1 | .678^a^ | .460 | .455 | 9659.265 |

| a. Predictors: (Constant), IgG3 concentration, IgG1 concentration |
| --- |

| **ANOVA^a^** | | | | | | |
| --- | --- | --- | --- | --- | --- | --- |
| Model | | Sum of Squares | df | Mean Square | F | Sig. |
| 1 | Regression | 16904137301.158 | 2 | 8452068650.579 | 90.589 | .000^b^ |
|  | Residual | 19873196565.837 | 213 | 93301392.328 |  |  |
|  | Total | 36777333866.995 | 215 |  |  |  |

| a. Dependent Variable: Total IgG concentration |
| --- |
| b. Predictors: (Constant), IgG3 concentration, IgG1 concentration |

| **Coefficients^a^** | | | | | | |
| --- | --- | --- | --- | --- | --- | --- |
| Model | | Unstandardized Coefficients | | Standardized Coefficients | t | Sig. |
|  |  | B | Std. Error | Beta |  |  |
| 1 | (Constant) | 4169.704 | 965.752 |  | 4.318 | .000 |
|  | IgG1 concentration | .225 | .037 | .309 | 6.121 | .000 |
|  | IgG3 concentration | 5.375 | .460 | .589 | 11.684 | .000 |

| **Coefficients^a^** | | | |
| --- | --- | --- | --- |
| Model | | Collinearity Statistics | |
|  |  | Tolerance | VIF |
| 1 | (Constant) |  |  |
|  | IgG1 concentration | .998 | 1.002 |
|  | IgG3 concentration | .998 | 1.002 |

| a. Dependent Variable: Total IgG concentration |
| --- |

ASUTUARE V2 (RAINY SEASON) – PFS230

| **Model Summary** | | | | |
| --- | --- | --- | --- | --- |
| Model | R | R Square | Adjusted R Square | Std. Error of the Estimate |
| 1 | .351^a^ | .124 | .111 | 6148.669 |

| a. Predictors: (Constant), IgG3 concentration, IgG1 concentration |
| --- |

| **ANOVA^a^** | | | | | | |
| --- | --- | --- | --- | --- | --- | --- |
| Model | | Sum of Squares | df | Mean Square | F | Sig. |
| 1 | Regression | 740637158.282 | 2 | 370318579.141 | 9.795 | .000^b^ |
|  | Residual | 5255052147.295 | 139 | 37806130.556 |  |  |
|  | Total | 5995689305.577 | 141 |  |  |  |

| a. Dependent Variable: Total IgG concentration |
| --- |
| b. Predictors: (Constant), IgG3 concentration, IgG1 concentration |

| **Coefficients^a^** | | | | | | |
| --- | --- | --- | --- | --- | --- | --- |
| Model | | Unstandardized Coefficients | | Standardized Coefficients | t | Sig. |
|  |  | B | Std. Error | Beta |  |  |
| 1 | (Constant) | 3572.269 | 771.471 |  | 4.630 | .000 |
|  | IgG1 concentration | 1.408 | .478 | .235 | 2.945 | .004 |
|  | IgG3 concentration | 2.267 | .771 | .235 | 2.940 | .004 |

| **Coefficients^a^** | | | |
| --- | --- | --- | --- |
| Model | | Collinearity Statistics | |
|  |  | Tolerance | VIF |
| 1 | (Constant) |  |  |
|  | IgG1 concentration | .987 | 1.014 |
|  | IgG3 concentration | .987 | 1.014 |

| a. Dependent Variable: Total IgG concentration |
| --- |

OBOM V2 (DRY SEASON) – MSP3

| **Model Summary** | | | | |
| --- | --- | --- | --- | --- |
| Model | R | R Square | Adjusted R Square | Std. Error of the Estimate |
| 1 | .068^a^ | .005 | -.006 | 43534.881 |

| a. Predictors: (Constant), Obv2 IgG3m, Obv2 IgG1m |
| --- |

| **ANOVA^a^** | | | | | | |
| --- | --- | --- | --- | --- | --- | --- |
| Model | | Sum of Squares | df | Mean Square | F | Sig. |
| 1 | Regression | 1615555076.872 | 2 | 807777538.436 | .426 | .654^b^ |
|  | Residual | 346837308153.752 | 183 | 1895285836.906 |  |  |
|  | Total | 348452863230.624 | 185 |  |  |  |

| a. Dependent Variable: Obv2IgGm |
| --- |
| b. Predictors: (Constant), Obv2 IgG3m, Obv2 IgG1m |

| **Coefficients^a^** | | | | | | | |
| --- | --- | --- | --- | --- | --- | --- | --- |
| Model | | Unstandardized Coefficients | | Standardized Coefficients | t | Sig. | Collinearity Statistics |
|  |  | B | Std. Error | Beta |  |  | Tolerance |
| 1 | (Constant) | 38808.383 | 5090.436 |  | 7.624 | .000 |  |
|  | Obv2 IgG1m | -3.124 | 6.685 | -.041 | -.467 | .641 | .712 |
|  | Obv2 IgG3m | 2.972 | 3.221 | .081 | .923 | .357 | .712 |

| **Coefficients^a^** | | |
| --- | --- | --- |
| Model | | Collinearity Statistics |
|  |  | VIF |
| 1 | (Constant) |  |
|  | Obv2 IgG1m | 1.404 |
|  | Obv2 IgG3m | 1.404 |

| a. Dependent Variable: Obv2IgGm |
| --- |

OBOM V3 (RAINY SEASON) – MSP3

| **Model Summary** | | | | |
| --- | --- | --- | --- | --- |
| Model | R | R Square | Adjusted R Square | Std. Error of the Estimate |
| 1 | .845^a^ | .714 | .709 | 11706.702 |

| a. Predictors: (Constant), Obv3IgG3m, Obv3IgG1m |
| --- |

| **ANOVA^a^** | | | | | | |
| --- | --- | --- | --- | --- | --- | --- |
| Model | | Sum of Squares | df | Mean Square | F | Sig. |
| 1 | Regression | 38067908711.225 | 2 | 19033954355.613 | 138.886 | .000^b^ |
|  | Residual | 15212202922.529 | 111 | 137046873.176 |  |  |
|  | Total | 53280111633.754 | 113 |  |  |  |

| a. Dependent Variable: Obv3IgGm |
| --- |
| b. Predictors: (Constant), Obv3IgG3m, Obv3IgG1m |

| **Coefficients^a^** | | | | | | | |
| --- | --- | --- | --- | --- | --- | --- | --- |
| Model | | Unstandardized Coefficients | | Standardized Coefficients | t | Sig. | Collinearity Statistics |
|  |  | B | Std. Error | Beta |  |  | Tolerance |
| 1 | (Constant) | 10300.669 | 1934.400 |  | 5.325 | .000 |  |
|  | Obv3IgG1m | 4.861 | .531 | .526 | 9.150 | .000 | .777 |
|  | Obv3IgG3m | 4.472 | .561 | .458 | 7.966 | .000 | .777 |

| **Coefficients^a^** | | |
| --- | --- | --- |
| Model | | Collinearity Statistics |
|  |  | VIF |
| 1 | (Constant) |  |
|  | Obv3IgG1m | 1.286 |
|  | Obv3IgG3m | 1.286 |

| a. Dependent Variable: Obv3IgGm |
| --- |

OBOM V2 (DRY SEASON) – PFS230

| **Model Summary** | | | | |
| --- | --- | --- | --- | --- |
| Model | R | R Square | Adjusted R Square | Std. Error of the Estimate |
| 1 | .565^a^ | .319 | .313 | 20218.354 |

| a. Predictors: (Constant), IgG3 concentration, IgG1 concentration |
| --- |

| **ANOVA^a^** | | | | | | |
| --- | --- | --- | --- | --- | --- | --- |
| Model | | Sum of Squares | df | Mean Square | F | Sig. |
| 1 | Regression | 45742533483.620 | 2 | 22871266741.810 | 55.950 | .000^b^ |
|  | Residual | 97698860404.182 | 239 | 408781842.695 |  |  |
|  | Total | 143441393887.802 | 241 |  |  |  |

| a. Dependent Variable: Total IgG concentration |
| --- |
| b. Predictors: (Constant), IgG3 concentration, IgG1 concentration |

| **Coefficients^a^** | | | | | | |
| --- | --- | --- | --- | --- | --- | --- |
| Model | | Unstandardized Coefficients | | Standardized Coefficients | t | Sig. |
|  |  | B | Std. Error | Beta |  |  |
| 1 | (Constant) | 8377.958 | 2037.971 |  | 4.111 | .000 |
|  | IgG1 concentration | .387 | .061 | .346 | 6.328 | .000 |
|  | IgG3 concentration | 3.483 | .505 | .377 | 6.901 | .000 |

| **Coefficients^a^** | | | |
| --- | --- | --- | --- |
| Model | | Collinearity Statistics | |
|  |  | Tolerance | VIF |
| 1 | (Constant) |  |  |
|  | IgG1 concentration | .953 | 1.049 |
|  | IgG3 concentration | .953 | 1.049 |

| a. Dependent Variable: Total IgG concentration |
| --- |

OBOM V3 (RAINY SEASON) – PFS230

| **Model Summary** | | | | |
| --- | --- | --- | --- | --- |
| Model | R | R Square | Adjusted R Square | Std. Error of the Estimate |
| 1 | .136^a^ | .018 | .003 | 16381.367 |

| a. Predictors: (Constant), IgG3 concentration, IgG1 concentration |
| --- |

| **ANOVA^a^** | | | | | | |
| --- | --- | --- | --- | --- | --- | --- |
| Model | | Sum of Squares | df | Mean Square | F | Sig. |
| 1 | Regression | 650281596.825 | 2 | 325140798.412 | 1.212 | .301^b^ |
|  | Residual | 34617045140.168 | 129 | 268349187.133 |  |  |
|  | Total | 35267326736.992 | 131 |  |  |  |

| a. Dependent Variable: Total IgG concentration |
| --- |
| b. Predictors: (Constant), IgG3 concentration, IgG1 concentration |

| **Coefficients^a^** | | | | | | |
| --- | --- | --- | --- | --- | --- | --- |
| Model | | Unstandardized Coefficients | | Standardized Coefficients | t | Sig. |
|  |  | B | Std. Error | Beta |  |  |
| 1 | (Constant) | 11493.516 | 2209.300 |  | 5.202 | .000 |
|  | IgG1 concentration | .180 | .363 | .043 | .496 | .620 |
|  | IgG3 concentration | 1.015 | .692 | .128 | 1.466 | .145 |

| **Coefficients^a^** | | | |
| --- | --- | --- | --- |
| Model | | Collinearity Statistics | |
|  |  | Tolerance | VIF |
| 1 | (Constant) |  |  |
|  | IgG1 concentration | 1.000 | 1.000 |
|  | IgG3 concentration | 1.000 | 1.000 |

| a. Dependent Variable: Total IgG concentration |
| --- |
