# Supplementary material for: Stage-specific Plasmodium falciparum immune responses in afebrile adults and children living in the Greater Accra Region of Ghana
Source: Malar J. 2020 Feb 10;19:64. doi: 10.1186/s12936-020-3146-7 (PMC7011432; doi:10.1186/s12936-020-3146-7)
Supplement: Supplementary file 3 — Additional file 3. Correlation analysis of antibody responses obtained at Obom with age and parasite density. [file 12936_2020_3146_MOESM3_ESM.docx]

**Additional file 3**. Correlation analysis of antibody responses obtained at Obom with age and parasite density.

OBOM DRY SEASON MSP3 CORRELATION COEFFICIENTS

|  | Obv2 age | Obv2 PD | Obv2 IgG | Obv2 IgG1 | Obv2 IgG3 | Obv2 IgM |
| --- | --- | --- | --- | --- | --- | --- |
| Obv2 age |  | -0.032 | 0.044 | 0.151 | 0.263 | 0.089 |
| OBv2 PD | -0.032 |  | -0.024 | 0.050 | 0.115 | -0.102 |
| Obv2IgG | 0.044 | -0.024 |  | -0.046 | 0.091 | 0.355 |
| Obv2 IgG1 | 0.151 | 0.050 | -0.046 |  | 0.643 | 0.064 |
| Obv2 IgG3 | 0.263 | 0.115 | 0.091 | 0.643 |  | 0.036 |
| Obv2 IgM | 0.089 | -0.102 | 0.355 | 0.064 | 0.036 |  |

P-VALUES FOR CORRELATION COEFFICIENTS (OBOM DRY SEASON MSP3)

|  | Obv2 age | Obv2 PD | Obv2 IgG | Obv2 IgG1 | Obv2 IgG3 | Obv2 IgM |
| --- | --- | --- | --- | --- | --- | --- |
| Obv2 age |  | 0.616 | 0.492 | 0.023 | 0.000 | 0.174 |
| Obv2 PD | 0.616 |  | 0.706 | 0.454 | 0.095 | 0.116 |
| Obv2 IgG | 0.492 | 0.706 |  | 0.488 | 0.190 | 0.000 |
| Obv2 IgG1 | 0.023 | 0.454 | 0.488 |  | 0.000 | 0.356 |
| Obv2 IgG3 | 0.000 | 0.095 | 0.190 | 0.000 |  | 0.612 |
| Obv2 IgM | 0.174 | 0.116 | 0.000 | 0.356 | 0.612 |  |

OBOM DRY SEASON PFS230 CORRELATION COEFFICIENTS

|  | Obv2 age | Obv2 PD | Obv2 IgG | Obv2 IgG1 | Obv2 IgG3 | Obv2 IgM |
| --- | --- | --- | --- | --- | --- | --- |
| Obv2 age |  | -0.033 | 0.048 | 0.045 | 0.088 | 0.027 |
| Obv2 PD | -0.033 |  | 0.016 | 0.090 | 0.080 | 0.013 |
| Obv2 IgG | 0.048 | 0.016 |  | 0.364 | 0.454 | 0.346 |
| Obv2 IgG1 | 0.045 | 0.090 | 0.364 |  | 0.298 | 0.352 |
| Obv2 IgG3 | 0.088 | 0.080 | 0.454 | 0.298 |  | 0.355 |
| Obv2 IgM | 0.027 | 0.013 | 0.346 | 0.352 | 0.355 |  |

P-VALUES FOR CORRELATION COEFFICIENTS (OBOM DRY SEASON PFS230)

|  | Obv2age | OBV2PD | Obv2IgG 2 | Obv2 IgG1 2 | Obv2 IgG3 2 | Obv2 IgM 2 |
| --- | --- | --- | --- | --- | --- | --- |
| Obv2age |  | 0.598 | 0.440 | 0.468 | 0.165 | 0.674 |
| OBV2PD | 0.598 |  | 0.798 | 0.157 | 0.217 | 0.844 |
| Obv2IgG | 0.440 | 0.798 |  | 0.000 | 0.000 | 0.000 |
| Obv2 IgG1 | 0.468 | 0.157 | 0.000 |  | 0.000 | 0.000 |
| Obv2 IgG3 | 0.165 | 0.217 | 0.000 | 0.000 |  | 0.000 |
| Obv2 IgM | 0.674 | 0.844 | 0.000 | 0.000 | 0.000 |  |

OBOM RAINY SEASON MSP3 CORRELATION COEFFICIENTS

|  | Obv3 age | Obv3 PD | Obv3 IgG | Obv3 IgG1 | Obv3 IgG3 | Obv3 IgM |
| --- | --- | --- | --- | --- | --- | --- |
| Obv3 age |  | -0.264 | 0.222 | 0.173 | 0.257 | 0.085 |
| Obv3 PD | -0.264 |  | 0.025 | 0.043 | -0.003 | 0.010 |
| Obv3 IgG | 0.222 | 0.025 |  | 0.810 | 0.741 | 0.350 |
| Obv3 IgG1 | 0.173 | 0.043 | 0.810 |  | 0.563 | 0.235 |
| Obv3 IgG3 | 0.257 | -0.003 | 0.741 | 0.563 |  | 0.358 |
| Obv3 IgM | 0.085 | 0.010 | 0.350 | 0.235 | 0.358 |  |

P-VALUES FOR CORRELATION COEFFICIENTS (OBOM RAINY SEASON MSP3)

|  | Obv3 age | Obv3 PD | Obv3 IgG | Obv3 IgG1 | Obv3 IgG3 | Obv3 IgM |
| --- | --- | --- | --- | --- | --- | --- |
| Obv3 age |  | 0.002 | 0.011 | 0.053 | 0.004 | 0.333 |
| Obv3 PD | 0.002 |  | 0.781 | 0.636 | 0.975 | 0.910 |
| Obv3 IgG | 0.011 | 0.781 |  | 0.000 | 0.000 | 0.000 |
| Obv3 IgG1 | 0.053 | 0.636 | 0.000 |  | 0.000 | 0.008 |
| Obv3 IgG3 | 0.004 | 0.975 | 0.000 | 0.000 |  | 0.000 |
| Obv3 IgM | 0.333 | 0.910 | 0.000 | 0.008 | 0.000 |  |

OBOM RAINY SEASON PFS230 CORRELATION COEFFICIENTS

|  | Obv3age | OBV3PD | Obv3IgG 2 | Obv3IgG1 2 | Obv3IgG3 2 | Obv3IgM 2 |
| --- | --- | --- | --- | --- | --- | --- |
| Obv3age |  | -0.286 | 0.172 | -0.017 | 0.048 | 0.117 |
| OBV3PD | -0.286 |  | 0.092 | 0.103 | 0.069 | -0.121 |
| Obv3IgG | 0.172 | 0.092 |  | 0.210 | 0.379 | 0.298 |
| Obv3IgG1 | -0.017 | 0.103 | 0.210 |  | 0.138 | 0.052 |
| Obv3IgG3 | 0.048 | 0.069 | 0.379 | 0.138 |  | 0.208 |
| Obv3IgM | 0.117 | -0.121 | 0.298 | 0.052 | 0.208 |  |

P-VALUES FOR CORRELATION COEFFICIENTS (OBOM RAINY SEASON PFS230)

|  | Obv3 age | Obv3 PD | Obv3 IgG | Obv3 IgG1 | Obv3 IgG3 | Obv3 IgM |
| --- | --- | --- | --- | --- | --- | --- |
| Obv3 age |  | 0.000 | 0.017 | 0.823 | 0.581 | 0.173 |
| Obv3 PD | 0.000 |  | 0.204 | 0.171 | 0.425 | 0.158 |
| Obv3 IgG | 0.017 | 0.204 |  | 0.005 | 0.000 | 0.000 |
| Obv3 IgG1 | 0.823 | 0.171 | 0.005 |  | 0.115 | 0.556 |
| Obv3 IgG3 | 0.581 | 0.425 | 0.000 | 0.115 |  | 0.015 |
| Obv3 IgM | 0.173 | 0.158 | 0.000 | 0.556 | 0.015 |  |

Obv2, Obom dry season; Obv3, Obom rainy season; PD, parasite density
